# SOD1 in Amyotrophic Lateral Sclerosis: “Ambivalent” Behavior Connected to the Disease

**DOI:** 10.3390/ijms19051345

**Published:** 2018-05-03

**Authors:** Orietta Pansarasa, Matteo Bordoni, Luca Diamanti, Daisy Sproviero, Stella Gagliardi, Cristina Cereda

**Affiliations:** 1 Genomic and Post-Genomic Center, IRCCS Mondino Foundation, Pavia 27100, Italy; orietta.pansarasa@mondino.it (O.P.); matteo.bordoni@mondino.it (M.B.); daisy.sproviero@mondino.it (D.S.); stella.gagliardi@mondino.it (S.G.); 2 Department of Brain and Behavioral Sciences, University of Pavia, Pavia 27100, Italy; luca.diamanti@mondino.it; 3 General Neurology, IRCCS Mondino Foundation, Pavia 27100, Italy

**Keywords:** amyotrophic lateral sclerosis, SOD1 protein, nuclear re-localization, DNA damage

## Abstract

In 1993, Rosen and collaborators discovered that the gene encoding SOD1 has mutations in amyotrophic lateral sclerosis (ALS) patients; moreover, these mutations are found in the exon regions, suggesting that their toxic effects are the consequence of protein dysfunction with an increase of oxidative stress. While a clear genetic picture has been delineated, a more complex scenario has been ascribed to the SOD1 protein. On the one hand, some evidence sustains the hypothesis of an additionally toxic role for wild-type SOD1 (WT-SOD1) in the pathogenesis of sporadic ALS. On the other hand, our group identified a discrepancy among WT-SOD1 protein expression levels and mRNA in ALS sporadic patients, thus providing the hypothesis of a re-localization of the “missing” SOD1 in a different sub-cellular compartment, i.e., nucleus, or an aggregation/precipitation in the insoluble fraction. Moreover, our data also indicate an association between longer disease duration and higher amounts of soluble SOD1 within the nucleus, suggesting a possible defensive role of the protein in this compartment. Starting from this evidence, in this review we will attempt to resolve the “ambivalent” behavior of SOD1 in ALS disease and we will try to classify sporadic ALS patients according to a novel biological signature, i.e., SOD localization.

## 1. Introduction

Amyotrophic lateral sclerosis (ALS) is a fatal adult-onset neurodegenerative disorder characterized by the progressive loss of motor neurons in the brain, brainstem, and spinal cord, which culminates in paralysis and death within a few years of diagnosis. When symptoms appear first in the arms or legs, ALS is commonly referred to as “limbic onset”; when speech problems prevail instead, ALS is referred to as “bulbar onset”. Usually, death occurs within 3–5 years from disease onset mainly caused by respiratory paralysis, but extreme cases with patients’ survival >40 years have recently been reported [1]. The annual incidence of ALS is 2.1 per 100,000 people, with an estimated prevalence of 5.4 cases per a population of 100,000 [2].

New technologies for gene mapping have enabled the identification of nearly 30 genes in ALS pathogenesis. Starting from *SOD1*, whose discovery in 1993 made it the first ALS gene to be identified [3], at least 25 genes have been implicated in familial ALS (fALS), sporadic ALS (sALS), or both. Along with *SOD1*, *TARDBP* [4,5], *FUS* [6,7], *OPTN* [8], *VCP* [9,10], *UBQLN2* [11], *C9orf72* [12,13] and recently *TBK1* [14,15] have all been indicated as causes of ALS. sALS and fALS are clinically identical, and since gene mutations are also present in sALS cases, the disease can be suggested to be complex and multi-factorial.

This review will provide an examination of the *SOD1* gene structure and of the mechanisms which regulate SOD1 transcription; of the role of SOD1 protein, detrimental or defensive, according to its sub-cellular localization; and of SOD1 participation in cell-to-cell communication. Finally, we reviewed new and relevant therapies for a personalized medicine approach.

## 2. Superoxide Dismutase 1 (*SOD1*) Gene

**Figure 1:**
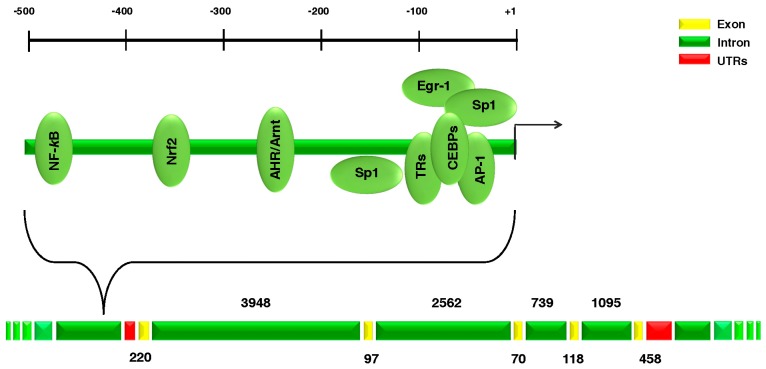
Structure of the *SOD1* gene.

The human *SOD1* gene (Entrez Gene ID 6647) is located on chromosome 21q22.11, and it codes for the monomeric SOD1 protein (153 amino acids, molecular weight 16 kDa). According to UCSC Genome Browser (GRCh37/hg19; available online: http://genome.ucsc.edu/), the *SOD1* gene is located from base pair 33,031,935 to base pair 33,041,241 with a genomic size of 9307 bp (Figure 1).

To date, over 180 different mutations have been described including single point mutations, deletions, insertions and truncation mutations throughout the five exons of the *SOD1* gene, pointing out that SOD1 toxic effects are the results of protein’s failures [16].

The most common mutations found in the *SOD1* gene are the D90A, the A4V and the G93A. The D90A (aspartic acid at codon 90 changed to alanine) is the most common mutation and can be inherited as both a dominant and a recessive trait, although in the majority of cases it is recessive [17]. The A4V (alanine at codon 4 changed to valine) is the most common (approximately 50%) ALS-causing mutation in the U.S. population [18]. The G93A (glycine 93 changed to alanine) is indeed a relatively rare mutation; nevertheless, it has been investigated very carefully, as it was the first mutation to be studied in a transgenic mouse model, where it is able to generate motor neuron syndrome [18].

Several transgenic murine lines for SOD1 have been generated, i.e., knockout mice deficient in SOD1 and mice bearing a human transgene of fALS-associated mutant *SOD1* [19,20]. For example, mice carrying the G85R mutation exhibit a reduced life span and display SOD1/ubiquitin-positive aggregates [21]. It is widely accepted that a hallmark of SOD1-associated ALS is the deposition of SOD1 into insoluble aggregates in motor neurons, probably due to a consequence of structural destabilisation and/or oxidative damage induced by gene mutations which in turn contribute to the misfolding and aggregation of SOD1 into neurotoxic species [22].

## 3. SOD1 Protein

Human Cu–Zn SOD1 is a 32 kDa, homo-dimeric metalloprotein, consisting of 153 aminoacids (molecular weight 16 kDa) and with the two subunits linked non-covalently [23]. SOD1 is mainly distributed in cytoplasm; however, it was found also in nucleus, lysosomes and mitochondria [24]. Its main function is to dismutate the free superoxide radicals (O_2_^•−^) into molecular oxygen (O_2_) along with the less reactive hydrogen peroxide (H_2_O_2_), thus eliminating the free radicals cause of oxidative stress [25].

Increased aggregation, dimer destabilization and oligomerization are all mechanisms proposed for mutant SOD1 toxicity, and each of which might not be mutually exclusive. Indeed, the presence of a mutation, oxidative stress, and anomalous metal binding, as well as other mechanisms to induce toxicity are initiated.

In regard to this, critical questions that remain unanswered include the following: the processes by which SOD1 transcription is regulated; the role of SOD1 in sALS patients, and how SOD1 participates in the cell-to-cell transmission of aggregates. Answering these crucial questions will ultimately help to defeat this devastating disease.

## 4. Transcriptional and Post-Transcriptional Regulation of SOD1

### 4.1. Trascriptional Regulation

*SOD1* induction is fine-tuned modulated by intracellular events involving both positive and negative regulatory elements acting jointly. Many studies have been performed to identify the position and the functional relevance of the *SOD1 cis-*acting elements and the corresponding *trans*-acting factors.

The CCAAT/Enhancer Binding Proteins (C/EBPs), is a family of transcription factors (TFs) characterized by a highly conserved, basic leucine zipper (bZIP) domain at the C-terminus. The C/EBP-related factors are required for SOD1 constitutive expression; both C/EBPα and C/EBPβ interact with the CAAT box and play roles on SOD1 basal transcription [26]. Moreover, the transcription factor CCAAT/enhancer binding protein delta (CEBPD, C/EBPδ, NF-IL6β) is involved in the regulation of human SOD1 transcription [27].

The Specificity Protein 1 (Sp1) is a ubiquitously expressed C2H2-type zinc finger-containing DNA binding protein; it activates *SOD1* promoter activity, thus suggesting that ectopic Sp1 overexpression markedly increased *SOD1*-basal promoter activity. Because of its ubiquity, Sp1 could interact with other activators or repressors of *SOD1* expression [28].

The Early Growth Response-1 (Egr1) is a nuclear phosphoprotein of 80 kDa that regulates transcription and belongs to the family of early response genes. Minc and co-workers (1999) using HeLa cells, reported an increase in SOD1 mRNA level after phorbol-12-myristate-13-acetate (PMA) treatment. They also identified a region, between nucleotides −59 and −48, that displays noncanonical consensus recognition sequences for Sp1, bound in a constitutive manner, and by Egr1 as a response to PMA exposure [29].

The C/EBP consensus element and the Sp1/Egr1 sequence partially overlap, thus supporting the existence of an interconnected network among these TFs to regulate *SOD1* gene expression.

The Activating Protein 1 (AP-1) is a homo- or heterodimeric TF made by proteins from Jun, Fos, and Maf subfamilies. AP-1 represses SOD1 transcription by sequestrating co-activators, such as Sp1, rather than interacting directly with the *SOD1* gene promoter [28]. Moreover, neuronal nitric oxide synthase (nNOS) over-expression causes downregulation of SOD1 mRNA, protein, and activity levels, probably because of a decreased binding between the Sp1 and *SOD1* promoter, caused by nNOS interaction with Sp1 [30].

The Aryl Hydrocarbon Receptor (AHR) is a ligand-activated TF belonging to the helix-loop-helix (bHLH) family. Cho and collaborators (2001) reported an increased expression of SOD1 mRNA and protein expression levels after the exposure of human HepG2 and HeLa cells to the 2,3,7,8-tetrachlorodibenzo-p-dioxin (TCDD), an environmental contaminant which interacts with AHR. The authors observed the presence of a xenobiotic responsive element in the 5′ flanking region of the human *SOD1* gene (between −255 and −238 from the transcription start site), which seems to be responsible for the induction by TCDD [31].

The Nuclear Factor-KappaB (NF-κB) points to a family of five TFs (p50, p52, RelA/p65, c-Rel, and RelB), all containing the Rel homology domain (RHD) at the N-terminus and acting as homo- and heterodimeric DNA binding complexes [32]. Rojo and colleagues (2004) identified a p65-NF-κB binding site in the human *SOD1* promoter (GGTAAGTCCC), and they demonstrated that Akt-activated NF-κB presents enhanced binding to this sequence, mediating the upregulation of *SOD1* expression [33].

The Thyroid Hormone Receptors (TRs) are encoded by the *TRα* and *TRβ* genes and are ligand-dependent TFs; they bind thyroid hormones (THs) and TH-response elements (TREs) that are located in the promoters of target genes. Santos et al., in 2006, identified a TH inhibitory element between −157 and +17 of the human *SOD1* promoter and established that Triiodothyronine (T3) exposure reverses the induction of SOD1 transcription triggered by paraquat, a ROS-producing element, and PMA agents. On the contrary, unliganded TRs significantly upregulate the *SOD1* promoter [34].

The Nuclear Factor E2-Related Factor 2 (Nrf2) is a Cap′n′collar (Cnc) TF that regulates the expression and the corresponding induction of defensive genes encoding phase II detoxifying enzymes and antioxidant proteins. Its activity is regulated by the Kelch-like ECH-associated protein 1 (Keap1). The dissociation from Keap1 is essential for Nrf2 translocation into the nucleus where it binds to the *cis*-acting antioxidant/electrophile responsive element (ARE/EpRE), and activates the transcription of genes involved in the detoxification from xenobiotics, ROS scavenging, and regulation of intracellular redox homeostasis [35,36]. The ARE/EpRE was first identified between −356 and −330 base pairs from the transcription start site (TSS) of the human *SOD1* gene promoter [37]. Moreover, the induction of *SOD1* gene transcription in human HepG2 hepatoma cells after dioxin TCDD treatment activates the Nrf2 signalling promoter [37]. Finally, low-dose and nontoxic proteasome inhibitors enhance mRNA and protein expression of SOD1 in human endothelial and vascular smooth muscle cells by Nrf2-mediated transcriptional induction [38].

In 2015, for the first time, our group conducted investigations to see if the overexpression of *SOD1* could be associated with the transcriptional regulation through Nrf2-mediated transcriptional induction [39]. Briefly, we demonstrated that after H_2_O_2_ treatment, the *cis*-acting ARE sequence in the *SOD1* promoter recruits different TFs and co-factors which, under oxidative stimuli, work together to modulate SOD1 mRNA induction. Our data indicate that the *SOD1* promoter is not a canonical downstream target of the Nrf2 pathway and that Nrf2 acts as a “constitutive” TF binding to the *SOD1* ARE sequence even without stimuli. On the other side, H_2_O_2_ treatment seems to promote changes in the chromatin conformation, with variations in the relationship between the identified TFs [39]. Dell’Orco and collaborators thus concluded that the increase in *SOD1* gene expression induced by H_2_O_2_ treatment is the consequence of an intricate process concerning the activation of a transcriptional mechanism involving PolII and other mechanisms (i.e., chromatin conformational changes and epigenetic variations), which cooperate to control the rate between de novo protein synthesis, mRNA and protein stabilization [39]. A central question remains to identify TFs directly involved in SOD1 transcriptional induction under oxidative stimuli and to shed light on different mechanisms modulating SOD1 transcription.

### 4.2. Post-Trascriptional Regulation

Post-transcriptional control represents a definite and accurate regulatory mechanism for gene expression which can modify protein expression levels according to extracellular stimuli. Post-transcriptional regulation of mRNAs provides opportunities by which gene expression could be rapidly modulated. Indeed, mRNA processing and nuclear export, mRNA stability, translational efficiency, and microRNA-dependent modulation create a composite intracellular set-up determining the overall levels of specific mRNAs. Most mRNA regulatory elements are located within the 5′ and 3′UTRs; the 5′UTR is mostly involved in controlling mRNA translation [40], while the 3′UTR regulates various steps of mRNA metabolism and stability. In 1995, Kilk and colleagues first identified two species of SOD1 mRNA with different 3′UTR lengths, which produce in-vitro different amounts of SOD1 protein [41]. Cells transfected with cDNA containing the long 3′UTR produce three times more SOD1 protein than cells transfected with a cDNA presenting a deletion of the last 185 bp from the 3′UTR. The authors concluded that the capability of the long mRNA to produce more SOD1 enzyme may depend on specific sequences located in the 3′UTR, and they identified different AREs in this region: AUUUA, CUUUA, AUUUG, GUUUUA, AUUUU, and AUUUC [41]. AREs are also considered the docking sites for different RNA binding proteins (RBPs); among which ELAV or (Hu) proteins play a crucial role [42]. Milani and co-authors, in 2013, focused on the regulation of SOD1 mRNA levels through a post-transcriptional mechanism mediated by ELAV proteins [43]. By means of a neuroblastoma cell line (SH-SY5Y) treated with H_2_O_2_ and peripheral blood mononuclear cells (PBMCs), obtained from sALS patients, the authors demonstrated that ELAV proteins bind to SOD1 mRNA, increasing its stability and/or translation. A physical relation between ELAVs and SOD1 mRNA was demonstrated and a positive influence of this binding on SOD1 expression under oxidative stress was proposed. The authors also revealed the activation of ELAVs in both PBMCs and nervous tissues from sALS patients compared to healthy controls, thus suggesting that this event could be a consistent mechanism to the disease. Interestingly, increased mRNA level seems to be related to a de novo protein synthesis; indeed, the authors showed enhanced protein levels in both the cellular model of oxidative stress and in the motor cortex of post-mortem tissues from sALS patients compared to healthy controls [43].

## 5. Toxic Properties of SOD1

As previously stated, the common hallmark of neurodegenerative disorders, including ALS, is the presence of misfolded protein aggregates in specific regions of the nervous system; hence these diseases are also identified as “protein misfolding disorders” [44].

Thanks to the evidence of a 32 kDa endogenously modified SOD1 species in the spinal cord extracts of sALS patients, Gruzman and colleagues in 2007 first suggested the hypothesis of the involvement of altered WT-SOD1 in sALS pathogenesis [45]. Indeed, the authors provided evidence that a single crosslinked protein species of SOD1 is common in both fALS and sALS. In the same year, Ezzi and co-authors also demonstrated that WT-SOD1 can gain, by means of oxidation, many of the toxic properties of ALS-linked mutant SOD1 [46]. The presence of misfolded SOD1, in the form of small aggregates in the nuclei of glial cells (mostly astocytes) of the spinal cord from ALS patients, was also demonstrated by Forsberg et al. [47].

In addition, Sau and co-authors (2007) [48] reported that, in the nucleus of motoneurons of transgenic mice and in immortalized motoneurons expressing either human WT-SOD1 or mutant SOD1 (G93A-SOD1), the levels of G93A-SOD1 are reduced in comparison to those of WT-SOD1. The loss of WT-SOD1 function in the nucleus may cause an alteration of the genomic DNA consequent to a reactive species attack. In turn, DNA damage may be induced, and finally the protein expression profile in motoneurons could be altered [48]. More recently, our group described the presence of hyper-oxidized aggregated SOD1 in lymphoblasts cell lines (LCLs) from a subset of sALS patients with bulbar onset [49]. We showed that WT-SOD1 is post-translationally modified and iper-oxidized in sALS patients, and that through this oxidation, altered SOD1 gains toxic properties similar to those due to disease-causing genetic mutations in patient-derived cells [49]. Guareschi et al., evidencing that only 30% of sALS involves iperOxSOD1, further highlight the concept that, similarly to fALS (with different genetic mutations), sALS could also be grouped according to different etiologies. Finally, these data could encourage the design of specific SOD1-based therapies in sALS and could identify biomarkers to sub-group various forms of sALS patients [49].

## 6. New Protective Function of Nuclear SOD1

In 2010, we reported an over-expression of SOD1 mRNA in PBMCs obtained from sALS patients [50], which apparently opposes the unchanged SOD1 protein expression level in the cytoplasm [51,52]. Thus, we questioned the discrepancy between protein and mRNA expression levels, i.e., “Where do we localize the missing SOD1?” and “What is the function of the re-localized SOD1?”

We answered the first question in 2013, when we demonstrated that the missing SOD1 translocates and re-localizes in the nuclear compartment. Indeed, we proved that in PBMCs of a sub-group of sALS patients, SOD1 was present in the nuclear compartment at higher levels; while perinuclear/cytoplasm SOD1 aggregates were found in the remaining ALS subjects [53], thus suggesting a possible relationship between the presence of protein aggregates and SOD1 enrichment in the insoluble fraction. On the other side, its higher nuclear expression profile seems to be associated to its higher solubility. In this study, we also found a positive correlation between longer disease duration and higher amount of soluble SOD1 in the nucleus, implying a possible protective role of the protein in this compartment [53].

Although evidence of a nuclear function of SOD1 protein has not yet been indicated, potential activities in addition to its scavenging ability have been described. In a tumour cell line, SOD1 counteracts stressful conditions by favoring the binding of transcriptional factors to DNA and inducing the synthesis of proteins with a protective role [54]. Nuclear SOD1 was essential for viability in conditional SOD1 knockout cells from chicken DT40 and SOD1 depletion increased sister chromatid exchange frequency and the number of apurinic/apyrimidinic sites, thus pointing to a new nuclear SOD1 function as a guardian of the genome [55]. Nuclear localization of SOD1 also reduced DNA lesions caused by an increase of the superoxide anion [55]. Additionally, patients characterized by higher soluble nuclear SOD1 amount presented insoluble cytoplasmic SOD1 levels similar to controls. These data are also sustained by two works of Forsberg [47,56] that, using specific antibodies against misfolded SOD1, identified two different staining patterns of SOD1 in motor neurons of sALS patients. Specifically, cells with intranuclear SOD1 had fewer aggregates in the cytoplasm, while cells with high misfolded SOD1 in the perinuclear area were characterized by the absence of nuclear staining [47,56]. Thus, we proposed two distinct fates for SOD1: the protein could remain soluble and able to exert its function(s) shuttling in the nucleus, or could aggregate in the cytoplasm ending up in insoluble fraction. Taking together evidence from the literature [54,55] and our previous observations [53], we presume that the re-localization of SOD1 in the nuclear compartment and the consequent increased accumulation of nuclear soluble SOD1 represents a potential physiological defensive mechanism of the cells against stressor stimuli. In 2014, Tsang and colleagues [57] suggested that in yeast, SOD1 has a new function in controlling the response to oxidative stress stimuli. Moreover, the authors expressed the fact that H_2_O_2_ does not cause an increase in the cellular superoxide levels, and suggested that the function of SOD1, once in the nucleus, is unrelated to its physiological role of removing damaging superoxide anions [57].

Starting from our previous data and from the evidence obtained by Tsang and co-authors [57], we tried to answer the second question, i.e., “What is the function of the re-localized SOD1?” It is widely accepted that accurate information on the exact subcellular localization of a protein is critical for understanding its physiological and pathophysiological function. By analyzing PBMCs from sALS patients, we found a re-localization of SOD1 in the nucleus, thus supporting Tsang’s results. Sau and collaborators [48] demonstrated that cells expressing G93A-SOD1 present higher levels of DNA damage compared with cells expressing WT-SOD1, and they ascribed this to a possible loss of the nuclear protection. In our cohort of sALS patients, when SOD1 is localized mainly at the nuclear level, the Comet tail, used as an index of DNA damage, is very weak and small, indicating that possibly the presence of SOD1 in this cellular compartment has a protective role against DNA damage. On the other hand, when SOD1 aggregates at the perinuclear/cytoplasm level, Comet tails are highly prominent, thus identifying remarkable levels of genomic damage. Therefore, we can hypothesize that in a sub-group of sporadic ALS patients, nuclear SOD1 probably cross-talks with different mechanisms to coordinate DNA repair, cell cycle progression, transcription and apoptosis. Among these, the most credible seems to be the indirect stimulation of genes involved in DNA repair mechanism and this hypothesis fits well with Cereda’s [53] previous results, which strongly correlate with disease duration. Indeed, when SOD1 is present in the nucleus, the disease duration increases significantly.

**Figure 2:**
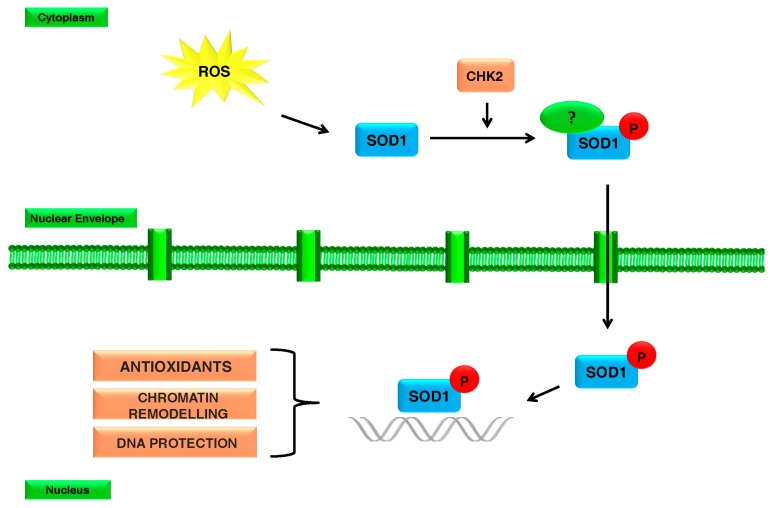
Schematic representation of SOD1 re-localization involving Chk2-mediated SOD1 phosphorylation.

Similar to Tsang and colleagues (2014) [57], who suggested that SOD1 acts as a nuclear transcription factor to regulate oxidative stress resistance, we also proposed a possible mechanism of action of SOD1. In particular, we hypothesized that in response to oxidative stress, SOD1 is prone to phosphorylation, especially on Thr residues in a Chk2-dependent manner, the human protein kinase homologue to the yeast Dun1 [57]. Phosphorylation seems to account for a SOD1 shift from the cytoplasm to the nucleus, where SOD1 could play a new protective function by binding the chromatin and by activating the transcription of DNA repair genes against oxidative DNA damage acting as a co-transcriptor factor (Figure 2). In particular, we can hypothesize that nuclear phospho-SOD1 could be able to place on the chromatin, while the cytoplasmic phospho-SOD1 could be involved in shuttling, in turn leading to SOD1 nuclear localization.

## 7. Intercellular Transmission of SOD1

Several studies have demonstrated that SOD1 participates in cell-to-cell propagation of aggregates [58,59], thus suggesting a “prion-like behavior” as a key mechanism underlying the aggregation and spreading of misfolded proteins [60]. One of the possible mechanisms proposed to explain cell-to-cell transmission of aggregates is the release of extracellular vesicles (EVs) [61]. EVs are spherical vesicles heterogeneous in size, classified into (i) apoptotic bodies (500–2000 nm in size) which released during cell death; (ii) microvesicles (MVs), of about 100–1000 nm in diameter, are shed from cells by outward protrusion (or budding) of a plasma membrane; (iii) exosomes (EXOs), with a diameter range of 40–150 nm, are produced by the fusion of multivesicular bodies with the cell membrane. They serve as highly specialized mechanisms of intercellular communication and once released by the donor cells, they may interact with target (recipient) cells by binding to cell surface receptors, membrane fusion, endosomal uptake and cargo extrusion [62]. EVs contain a rich cargo of proteins, coding and non-coding RNAs (also miRNA), lipids, DNA, and metabolites that are specifically sorted and that reflect the biological state of the cells’ origin.

EV secretion could be considered as an alternative route to eliminate aggregate-prone toxic proteins. The first evidence of an association between SOD1 and EXOs dates back to 2007, when Gomes and colleagues showed that NSC-34 motor neuron-like cells, transfected with WT-SOD1 and G93A-SOD1, release EXOs carrying WT and G93A-SOD1, respectively [63]. Moreover, in mice expressing mutant SOD1, Basso et al. (2013) [64] found an up-regulation of release of EXOs and an increased level of EXO-associated proteins in the medium of G93A-SOD1 astrocytes. This work also suggested that astrocytes employ the release of EXOs to clear cells from misfolded and possibly toxic proteins. Furthermore, the release of EXOs can exert a toxic effect on neighboring motor neurons, thus representing a way for disease spreading in neurodegenerative disorders [64,65].

Subsequently, Grad et al. reported that misfolded WT-SOD1 can be released via EXOs and uptaken in neuronal cells [66], thus indicating first the contribution of SOD1 in the propagation of misfolding, and second a common pathogenic mechanism between fALS and sALS [66].

Despite the increasing interest in the EVs field, until now some questions remain unanswered: “What is/are the mechanism/s leading to EV formation?” “Is the specificity of EV cargo fundamental for their function?”, “What are the proteins on the extracellular membrane of EVs that serve as receptors and mediate the identification of the right target cells?”

## 8. SOD1 as a Therapeutic Target in ALS Disease

The lack of a clear pathogenesis has strongly slowed the development of an effective therapy. With the exception of Riluzole, which prolongs survival for 2–3 months, and Edavarone, which decreases the rate of patient immobility, no treatments are currently available for ALS.

As such, the possibility of gene therapy has attracted particular attention in the context of fatal diseases such as ALS. For this purpose, SOD1 is a good target for therapy and is one of the main pathological hallmarks of the disease itself.

Gene therapy refers to the transfer of a therapeutic gene into a target tissue and the preservation of gene function for an adequate time.

The use of antisense oligonucleotides (ASO) has the potential to bypass poorly understood pathogenesis by targeting ALS directly at its origin: the transcripts [67]. ASOs are synthetic short nucleic acids that bind to RNA and they function in various ways, such as targeting RNA for degradation and preventing the translation of a specific RNA [68]. This idea applied to neurodegenerative disorders was first proposed in 2006 [69], but the first “real” trial based on SOD1-ASO was only published in 2013 by Miller and collaborators [70]. The authors described, in SOD1-mutated patients, that non-serious adverse events occurred after the intrathecal administration of ASO ISIS 333611 delivered to cerebrospinal fluid (CSF) [70]. The obtained data showed that a reduction of SOD1 concentration in brain tissue correlates with reduced SOD1 in the CSF; however, this study also had several limitations, in particular the administration of ASO ISIS 333611 was unable to cross the blood–brain barrier (BBB). At this point, Chen at al. conjugated SOD1-ASO with calcium phosphate lipid-coated nanoparticles to improve the delivery; so far, the data look very convincing in the case of zebrafish, but no experiments have been performed in an in vivo mouse model to hypothesize a possible use in ALS patients [71].

Together with the ASOs, small interfering RNA (siRNA) methods are also indicated as important RNA-targeted approaches in ALS. By injecting siRNA intramuscularly for delivery to spinal motor neurons through retrograde transport of adeno-associated virus (AAV), Miller and co-authors reported a reduction in SOD1 followed by a delayed loss of grip strength, in an SOD1G93A mouse model, thus supporting intramuscular injections of siRNA for fALS [72]. Indeed, in SOD1G93A mice, the intraspinal or intramuscular injections of lentiviruses with shRNA SOD1 increase retrograde transport, delay the onset of motor neuron symptoms (115%) and extend survival (77% of normal life) [73]. A delay in disease onset and progression has also been reported by other authors [74] in both SOD1G93A mice and in non-human primates. More recently, the combined use of intravenous and intracerebroventricular delivery of AAV10-U7-hSOD1 determined an increase in the survival rate of SOD1G93A mice injected at birth (92%) but also at 50 days of age (58%) and prevented the weight loss and the degeneration of the neuromuscular function [75].

Another potential powerful medical tool is represented by the CRISPR-Cas system [76]. Recently, using the CRISPR/Cas9 system, Wang and co-workers targeted genes to introduce the correction of mutations in induced pluripotent stem cells (iPSCs) obtained from fibroblasts of fALS SOD1 and FUS patients; these results were further supported by genomic DNA sequencing for SOD1 [77]. It is plausible that a combination between gene therapies which target simultaneously various disease mechanisms could provide additive positive effects in neuromuscular disorders. In particular, in a multi-systemic disease such as ALS, the possibility to co-modify pathways in different cell types seems to be crucial. For this purpose, viral vectors engineered with selective promoters could be combined with cell penetrating peptide (CPP)-conjugated ASOs, to synergistically target various genes in related and independent pathways through specific cell populations. The genetic suppression of the NF-κB pathway in microglia or shRNA-mediated knockdown of SOD1 in motor neurons and astrocytes via the systemic AAV9 administration determined improvements in survival, disease onset, and progression of mutant SOD1 mice. [78].

## 9. Conclusions

SOD1 has been broadly studied in different models since the discovery of its involvement in ALS disease.

However, critical questions remain unanswered. First, it is of great importance to understand the mechanisms involved in the up-regulation of *SOD1* gene transcription, and to extensively unravel the possible new role of SOD1 in ALS disease. On one hand, SOD1 could aggregate and misfold; on the other, when it re-localizes in the nuclear compartment, SOD1 acquires a new protective function mainly against DNA damage. Furthermore, understanding the mechanisms of prion-like protein misfolding and pathology propagation is critical for a comprehensive understanding of the etiology of ALS and for effective therapeutic development. It is obvious that all the “roles” of SOD1 are crucial to address the course of the disease; thus, in turn, helping in isolating the role of SOD1 in ALS disease could open new scenarios to develop new and promising therapies, i.e., ASO or the delivery of nanoparticles, for ALS in particular to design innovative pharmaco-therapies using ASOs, siRNAs and CRISPR to counteract ALS.
